# Relating evolutionary selection and mutant clonal dynamics in normal epithelia

**DOI:** 10.1098/rsif.2019.0230

**Published:** 2019-07-31

**Authors:** Michael W. J. Hall, Philip H. Jones, Benjamin A. Hall

**Affiliations:** 1 Wellcome Trust Sanger Institute, Hinxton CB10 1SA, UK; 2 MRC Cancer Unit, University of Cambridge, Hutchison-MRC Research Centre, Box 197, Cambridge Biomedical Campus, Cambridge CB2 0XZ, UK

**Keywords:** stem cells, DNA sequencing, oesophagus, cancer

## Abstract

Cancer develops from mutated cells in normal tissues. Whether somatic mutations alter normal cell dynamics is key to understanding cancer risk and guiding interventions to reduce it. An analysis of the first incomplete moment of size distributions of clones carrying cancer-associated mutations in normal human eyelid skin gives a good fit with neutral drift, arguing mutations do not affect cell fate. However, this suggestion conflicts with genetic evidence in the same dataset that argues for strong positive selection of a subset of mutations. This implies cells carrying these mutations have a competitive advantage over normal cells, leading to large clonal expansions within the tissue. In the normal epithelium, clone growth is constrained by the limited size of the proliferating compartment and competition with surrounding cells. We show that if these factors are taken into account, the first incomplete moment of the clone size distribution is unable to exclude non-neutral behaviour. Furthermore, experimental factors can make a non-neutral clone size distribution appear neutral. We validate these principles with a new experimental dataset showing that when experiments are appropriately designed, the first incomplete moment can be a useful indicator of non-neutral competition. Finally, we discuss the complex relationship between mutant clone sizes and genetic selection.

## 1. Introduction

Large-scale sequencing of cancer genomes has led to the discovery of many recurrently occurring genetic mutations that are potential ‘drivers’ of the disease [1–3]. Recently, however, a number of studies investigating normal tissues have found that many of these mutations are also present in apparently healthy tissue [4–9]. To understand tumorigenesis, it is therefore important to study the acquisition and spread of mutations in normal tissue.

**Figure 1.**
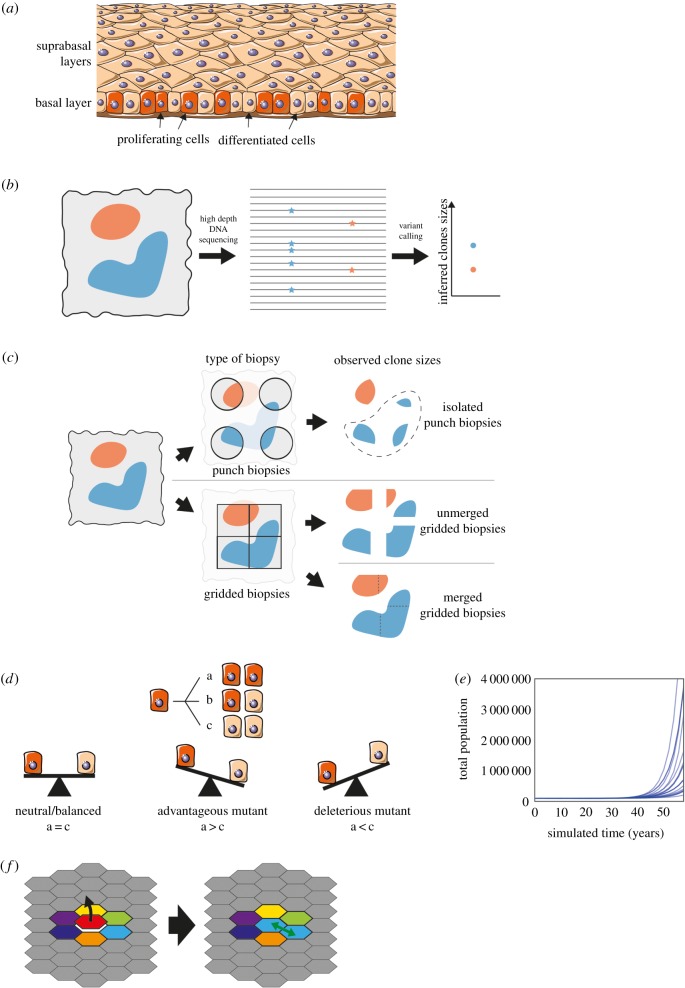
Data collection and cell dynamics. (*a*) Proliferation occurs in the basal layer of the epithelium. After differentiation, cells migrate through the suprabasal layers before being shed. Image from smart.servier.com, licensed under CC BY 3.0, edited from original. (*b*) DNA from a biopsy (left) containing mutant clones (red and blue) is sequenced and the VAF (proportion of reads containing the mutation, middle) used to infer clone sizes (right). (*c*) The method of taking biopsies can affect the observed mutant clone size distribution. Isolated punch biopsies (top) may not capture the entirety of a mutant clone; in the analysis in [11], clones that spanned multiple biopsies (shown in dashed area) were excluded. Ungapped gridded biopsies (bottom) enable the reconstruction of larger clone sizes. (*d*) The stochastic single progenitor model of cell dynamics. Each dividing cell (red) can produce two dividing cells (*a*), two non-dividing differentiated cells (brown) (*c*) or one of each type (*b*). In a homeostatic tissue or neutral clone, the probabilities of each symmetric division option are balanced (left). An advantageous mutation would increase the proportion of dividing cells produced (middle), and a deleterious mutation would increase the proportion of differentiated cells (right). Note that in the non-neutral case, the probabilities of each division type do not have to be fixed over time, but can depend on the cell context. (*e*) Simulation of the model shown in (*d*). If mutations introduce perpetual positive fate imbalances then the population will eventually explode. Total population of 20 simulations with mutations introducing only small fate imbalances drawn from *N* ∼ *N* (mean = 0.25%, std = 1.25%). (*f*) In the spatial Moran process, a differentiating cell (red) is replaced by the division of a neighbouring cell (light blue).

The most common human cancers are derived from squamous epithelia, which consist of layers of keratinocytes [10]. Cells are continually shed from the tissue surface and replaced by proliferation (figure 1*a*). The proliferating cells accumulate mutations over time [12]. If such clones persist within the otherwise normal tissue, they may acquire the additional genomic alterations that lead to cancer [12]. A key question is whether these large, persistent clones arise by neutral competition or are a consequence of cancer-associated mutations increasing the competitive fitness of mutant cells above that of wild-type cells. If the former, little can be done to alter the risk of cancers emerging from mutant clones in normal tissue. However, if the founding clones of cancers emerge by competitive selection, it is possible that interventions that alter the fitness of mutant cells may decrease cancer risk. A dataset of mutant clones detected in normal human eyelid skin appears to contain conflicting evidence supporting both neutral and non-neutral mutant cell dynamics [4,11]. This ‘paradox’ has yet to be resolved [13,14], leading to uncertainty over the somatic mutant cell dynamics in normal epithelial tissues [15]. This paper aims to unpick this apparent inconsistency.

The dataset in question is from a study of mutations in normal human eyelid skin epidermis [4]. DNA was extracted from small samples of epidermis. About 500 DNA molecules of each targeted gene were sequenced and compared to the genome of the same tissue donor (figure 1*b*). Somatic mutations were detected as altered sequences present in one or more samples [4]. The proportion of altered DNA reads containing a mutation, the variant allele fraction (VAF), was assumed to be proportional to the size of the mutant clone (figure 1*b*).

An analysis of inferred sizes of the mutant clones in the human eyelid data argues for neutral dynamics [11]. The observed clone sizes can be compared to the predictions of candidate mathematical models of cell dynamics to determine the best-fitting model [16]. Lineage tracing experiments in homeostatic, unmutated mouse epidermis and oesophagus suggest that these tissues are maintained by a single population of equipotent progenitor cells (figure 1*d*) [17–19]. The outcome of individual cell divisions is unpredictable, but on average, 50% of the progeny of dividing cells differentiate, exit the proliferative compartment and are eventually shed from the tissue while 50% remain to divide again. Such a balanced stochastic cell fate leads to wide variation in clone sizes, while the total cell population remains constant. Mutant clone sizes observed in the human eyelid skin have been compared to predictions from this neutral stochastic model [11]. The comparison is made using the first incomplete moment of the clone size distribution (Methods), which has been used in several studies to shed light on mutant clone growth dynamics [11,20–23]. In economics, the first incomplete moment is used to study inequality—the value of the incomplete moment at £*X* shows how much of the wealth is held by those with a fortune of £*X* or higher [24]. It has a similar role for the clone size analysis—it shows the proportion of the mutated cells that are in clones of size *x* or larger. Using the first incomplete moment has two advantages over using the clone size distribution directly. Firstly, the first incomplete moment reduces the fluctuations caused by low sample size [20] and secondly, it simplifies the comparison of data to the neutral theory. It is proposed that there will be a clear distinction between the logarithm of the first incomplete moment (LFIM) from neutral and non-neutral competition: neutral competition will lead to a straight line, whereas non-neutral competition will be indicated by a curved/kinked line. The neutral model predicts that the first incomplete moment of mutant clone sizes will have a negative exponential form [11]—where there are many small clones and few large clones. A deviation from the exponential shape could indicate non-neutral competition—some clones have expanded to take over more than their expected share of the tissue. To make it easier to see this deviation, we use the LFIM, which turns the exponential curve into a straight line. Non-neutral competition would then be indicated by a deviation from the straight line [11]. Using this criterion, the inferred mutant clone sizes from the human eyelid appear largely consistent with the neutral model [11].

However, the theory of neutral competition of cancer-associated mutations is incompatible with results from several mouse and human studies that observed non-neutral mutant expansions in normal epithelial tissues [18,20,25]. Furthermore, signs of non-neutral clonal competition in the eyelid mutational data can be detected using d*N*/d*S* analysis, a method from population genetics. This examines the ratio of protein-altering mutations (d*N*) to silent mutations (d*S*) for each gene [26]. Once relevant corrections have been applied, a d*N*/d*S* ratio of 1 is indicative of neutral behaviour. A d*N*/d*S* value of less than 1 indicates the mutated gene has a negative effect on the competitive fitness of mutant cells compared with normal cells. However, if a disruption of the protein provides a growth advantage to the cell, then the number of protein-altering mutations that expand to a clone of detectable size will be increased, leading to a d*N*/d*S* ratio greater than 1. Analysis of the human eyelid mutations found six of the 74 sequenced genes had significantly raised d*N*/d*S* ratios ranging from three to over 30, consistent with mutations in those genes driving clonal expansion [4]. Additionally, protein-altering missense mutations in some driver genes, e.g. *NOTCH1*, *NOTCH2* and *TP53*, were not randomly distributed but concentrated in functional domains. This suggests positive selection of function-altering mutations, which is also incompatible with neutrality [4].

Here, we show that in some conditions, *non-neutral* competition can produce a straight-line LFIM, and therefore, a straight LFIM alone is not a clear indicator of *neutral* dynamics. We can thus reconcile clone size distributions and positive genetic selection.

## 2. Results

We began by noting several important constraints that apply to the mutant clones in normal epithelia. Firstly, the cellular structure and composition of the tissue remains at least approximately constant. Secondly, the proliferative compartments of the epidermis and oesophageal epithelium contain few barriers to mutant clone expansion. In a tissue with a high burden of mutations like the human eyelid, this means that expanding clones will soon collide and compete with each other as well as with unmutated cells. These two constraints were not included in the mathematical model used in the first incomplete moment analysis of the human eyelid data [11], meaning mutant clones in this model with a growth advantage (figure 1*d*, middle) could expand without limit (figure 1*e*).

### 2.1. Spatial constraints alter clone size distributions of non-neutral mutations

To address the effect of clonal competition, we used a mathematical model drawn from the study of population genetics. We ran Moran-type [27] simulations of cell competition on a two-dimensional (2D) grid to represent the epidermis (figure 1*f*). Cells lost through differentiation are replaced by the division of a neighbouring cell (Methods), similar to behaviour observed in mouse epidermis [28]. During each division, there is a small chance that one of the daughter cells will acquire a mutation (Methods).

**Figure 2.**
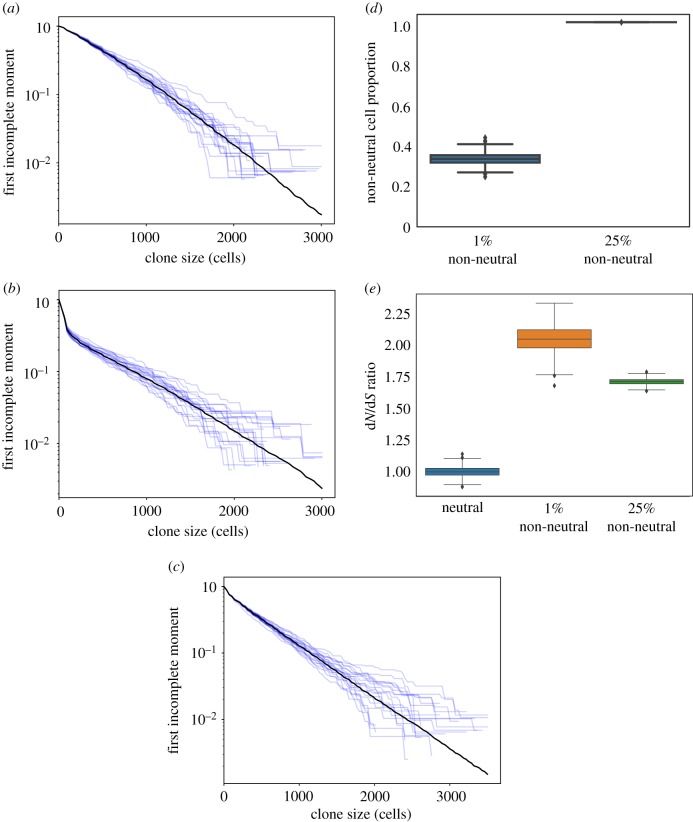
First incomplete moments of 2D simulations. (*a*–*c*) First incomplete moments of 2D simulations (Methods). The average of 1000 simulations is shown in black, a selection of 20 individual simulations is shown in blue. (*a*) Neutral simulations. (*b*) Simulations where 1% of mutations are non-neutral. A deviation from the straight line is seen at clone sizes of approximately 100 cells. (*c*) Simulations where 25% of mutations are non-neutral. (*d*) Proportion of cells at the end of the simulations with a fitness altered by non-neutral mutations. In the 25% non-neutral simulations, by the end of the simulation, almost the entirety of the tissue has been colonized by non-neutral mutant clones. (*e*) d*N*/d*S* values from the simulations shown in (*a*–*c*). To enable this calculation for the neutral simulations, a proportion of neutral mutations were labelled as non-neutral but did not affect cell fitness. (Online version in colour.)

Simulations of a 2D neutral model produced an approximately straight LFIM (figure 2*a*). A non-neutral spatial model in which a small proportion of mutations change the fitness of a cell (Methods) may deviate from a neutral appearance by curving away from the straight line (figure 2*b*). This was due to the contrast between the relatively large non-neutral clones and the smaller clones growing neutrally. Surprisingly, however, simulations with a higher proportion of non-neutral mutations may generate a straight line (figure 2*c*). This is because almost all of the simulated tissue is taken over by non-neutral clones (figure 2*d*). The only neutral mutations that persist are those that occur in clones with non-neutral mutations, carried as ‘passengers’. As all remaining clones exhibit similar behaviour, the LFIM is straightened. This shows that a straight-line LFIM does not necessarily imply neutral competition and is consistent with positive d*N*/d*S* ratios (figure 2*e*). We concluded that since there is a high burden of mutant clones in the eyelid, the tissue is likely to be extensively colonized by non-neutral mutant cells, contributing to the apparently neutral appearance of the clone size distribution.

### 2.2. Impact of sampling methods on measurement of clone size distributions

**Figure 3.**
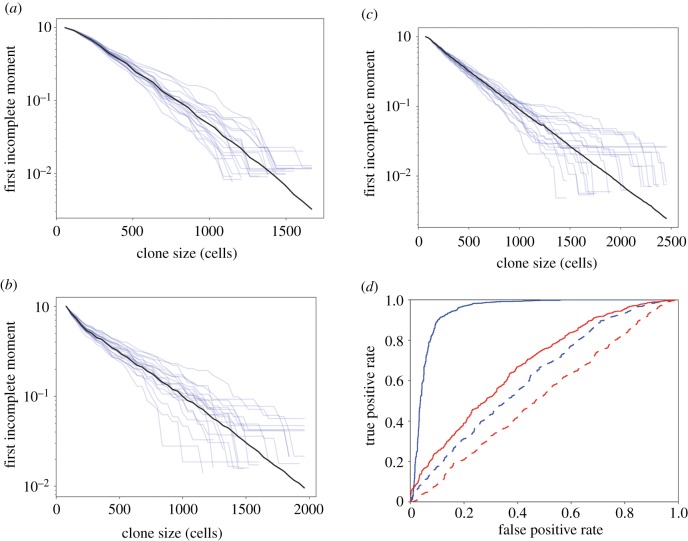
First incomplete moments of 2D simulations with biopsy sequencing. (*a*–*c*) The simulations from figure 2*a*–*c* respectively, with the effects of biopsy and sequencing. (*d*) ROC curves using *R*^2^ of the log first incomplete moment of the clone size distribution as the classifying statistic. Red, simulated biopsy and sequencing; blue, full data randomly subsampled to match biopsy plus sequencing sample sizes. Solid, 1% non-neutral; dash, 25% non-neutral. Area under the curve (AUC) is a measure of how successful the classifier is at distinguishing the two groups. A perfect classifier will have an AUC of 1. A random guess will have an AUC of 0.5. AUCs: full data, 1% non-neutral, 0.94; biopsy plus sequencing, 1% non-neutral, 0.68; full data, 25% non-neutral, 0.62; biopsy plus sequencing, 25% non-neutral, 0.52.

Another consideration that may impact the measurement of clonal size distributions and hence inference of mutant clone dynamics is experimental design. In the human eyelid study, spatially separated tissue samples were collected (figure 1*c*) [4]. The area of the sample defines an upper limit on the reliable estimation of clone size. In the eyelid experiment, the area of each sample was less than that of the largest clones. The lower limit of clone size detection is also related to the sample area, since mutations present in only a small fraction of the cells in the sample may not be detected due to the technical noise in DNA sequencing [29]. We simulated the combined effects of spaced samples in which only clones occupying 1% or more of the area of the sample can be detected (Methods). Figure 3*a*–*c* shows these effects on the first incomplete moments of the simulations from figure 2*a*–*c* respectively. The results lead us to conclude that these experimental factors may artefactually reduce a deviation of LFIM from a straight line caused by non-neutral competition (figures 2*b* and 3*b*).

### 2.3. Ability of logarithm of the first incomplete moment to resolve neutral competition versus selection

We next tested how well the LFIM could discriminate between the neutral and non-neutral simulations using the coefficient of determination, *R*^2^, to measure the straightness of a line, as in previous studies [11,23] (Methods). For the LFIM to be a successful indicator of neutrality, the neutral simulations need to have a higher *R*^2^ than the non-neutral simulations. Receiver operating characteristic (ROC) curves show the accuracy of the LFIM as a test of neutrality depended on both the underlying shape of a non-neutral clone size distribution and on the experimental sampling method (figure 3*d*). For example, using the LFIM was little better than a random guess (area under the curve, AUC = 0.52) when attempting to distinguish the neutral simulations from figure 3*a* from the non-neutral simulations in figure 3*c*, where the underlying shape of the non-neutral LFIM was largely straight (figure 2*c*) and the clones were measured using simulated DNA sequencing of spatially separated biopsies.

### 2.4. Human oesophageal mutant clone sizes demonstrate non-neutral growth

**Figure 4.**
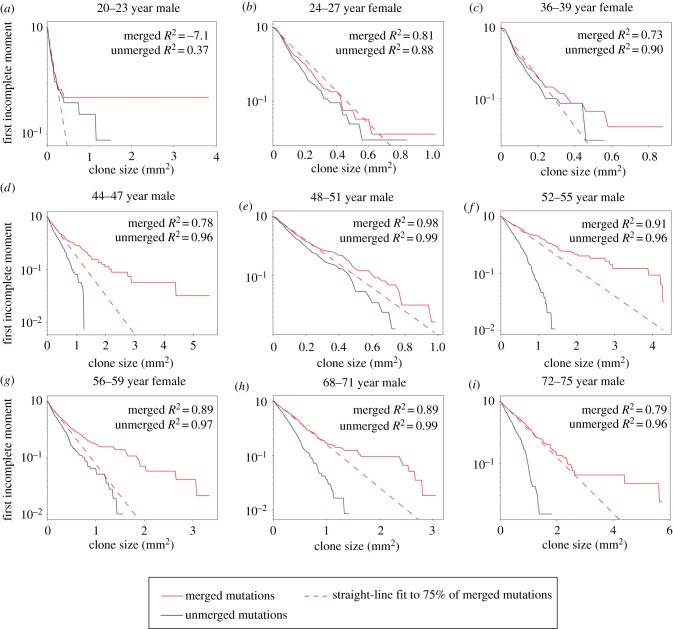
Normal human oesophagus. (*a*–*i*) First incomplete moments of the human oesophagus mutation data for the nine individuals in the study [5]. The clone sizes are either inferred from each 2 mm^2^ sample without merging (blue) or by using the gridded system to infer the size of mutations which span multiple samples using the methods of the original study [5] (red, solid). The extent of deviation from the straight line can be seen by comparing the data (solid) to the dashed red line, which shows a straight-line fit to the smallest 75% of clones in the merged case. Loss of heterozygosity (LOH) copy number changes were frequently found to co-occur with protein-altering *NOTCH1* mutations [5] and to obtain conservative estimates of clone sizes, we assume this is the case for all protein-altering *NOTCH1* mutations. All other mutations on chromosomes 1–22 were assumed to be heterozygous. *R*^2^ values can be negative because the line fitting is constrained to pass through the point (*m*, 1), where *m* was the smallest observed clone size. Ages given as a range for anonymization purposes.

To validate this analysis, we drew on a second experiment that measured mutant clones in normal human oesophageal epithelium [5]. d*N*/d*S* analysis revealed mutations in 14 of 74 genes sequenced were under significant positive selection [5]. This study used an ungapped sampling strategy in which the epithelium was cut into gridded arrays of samples which were then deep DNA sequenced, allowing the areas of clones that extend over multiple samples to be determined [5] (figure 1*c*). This key difference in design from the eyelid experiment allowed us to investigate the effect of sampling on the incomplete moment analysis of the eyelid data by comparing the gridded data with what would have happened if the oesophagus was sampled in the same manner as the eyelid skin (figure 1*c*). The LFIMs for clone sizes estimated from both sampling approaches are shown in figure 4. Taking figure 4*d* as an example, the gapped sampling approach results in an LFIM that fits well with a straight line (*R*^2^ = 0.96) and therefore appears consistent with neutral competition. However, if using the clone sizes based on a gridded approach, the LFIM deviates from the straight line (*R*^2^ = 0.78), suggesting non-neutral competition may have occurred in the tissue. For each of the nine individuals in the study, the LFIM exhibits a greater deviation from the straight line when using gridded samples than when using spaced samples.

**Figure 5.**
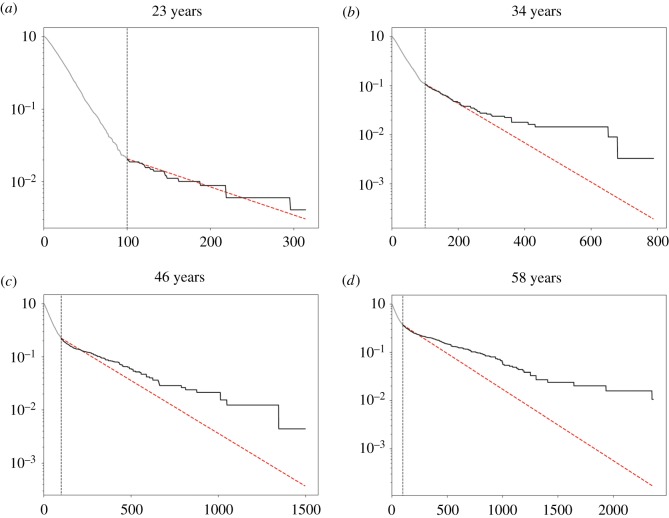
First incomplete moment over time. (*a*–*d*) Curves in the LFIM may only be visible after sufficient time has passed, allowing both fast- and slow-growing clones to reach large enough sizes to be detectable through sequencing. Examples of the first incomplete moment for a simulation of non-neutral competition are shown for four timepoints. One per cent of mutations are non-neutral with a fitness drawn from a normal distribution, *N* ∼*N* (mean = 0.1, std = 0.1). The vertical dashed line shows the detection limit, arbitrarily set at 100 cells. The section of the first incomplete moment that would not be visible due to the detection limit is shown in grey to the left of the line; the visible section is shown in black. The red dashed line is a straight-line fit to the smallest 75% of *visible* mutant clones. (Online version in colour.)

An intriguing pattern can be seen within the oesophageal data. With the exception of a few large clones, younger patients have less curved LFIMs, as shown by the deviation from a straight-line fit to the smallest 75% of clones (figure 4). Older individuals by contrast show a more distinct deviation from the line. This is consistent with simulations of the non-neutral competition (figure 5). At early timepoints, only the faster-growing non-neutral clones in the tail of the distribution are observed (figure 5*a*), leading to a straight-line LFIM. A curve is observed at later timepoints once the slower-growing clones reach a size large enough to be detected (figure 5*b*–*d*). Future work with an increased number of patients is required to confirm this apparent trend. Unfortunately, a recent publication providing further DNA sequencing of normal human oesophagus used isolated punch biopsies and is therefore not suitable for this kind of analysis [6].

### 2.5. Clone size as a marker of competitive selection of mutations

**Figure 6.**
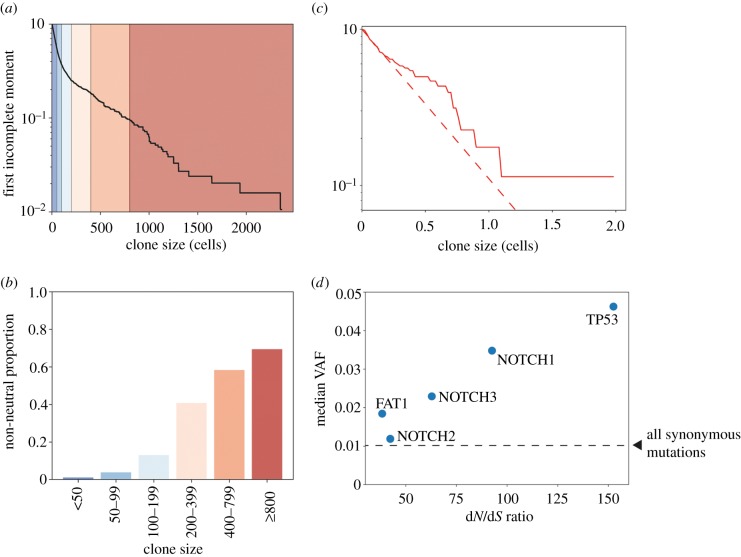
Clone size and selection. (*a*,*b*) Proportion of non-neutral clones in different size ranges. (*a*) The first incomplete moment of the clone size distribution from a simulation with 1% non-neutral mutations. Coloured regions correspond to ranges of clone sizes described in *b*. (*b*) Proportion of non-neutral clones in each clone size interval. Colours correspond to the regions shaded in *a*. (*c*) First incomplete moments of the human oesophagus mutation data for one individual, aged 72–75 [5], including only synonymous mutations and mutations in genes that are non-expressed (Methods). The synonymous mutation T125T in *TP53* was excluded as it has been found to affect splicing [12,26]. Clone sizes which extend across multiple samples are merged using the methods of the original study [5]. All mutations on chromosomes 1–22 were assumed to be heterozygous. The extent of deviation from the straight line can be seen by comparing the data (solid) to the dashed red line, which shows a straight-line fit to the smallest 75% of clones. (*d*) Median VAF for nonsense mutations in the five most significantly selected genes from the d*N*/d*S* analysis plotted against the d*N*/d*S* ratio for nonsense mutations. Combined results for all individuals in the study. The dashed line shows the median VAF of all synonymous mutations. Note that many of these synonymous mutations are likely to be passengers on non-neutral clonal expansions, and therefore, the line does not represent the median VAF of mutations that have grown solely under neutral drift. One-sided Mann–Whitney tests show that, aside from NOTCH2 (*p* = 0.06), nonsense mutant clones in the genes shown are significantly larger than synonymous mutant clones (*p* < 0.0001).

We have shown that clone size and LFIM alone cannot reliably classify clone sizes as neutral, due to a mixture of experimental limitations on the maximum and minimum sizes of clones and the fundamental effects of competition for space. In addition, where a curved LFIM is found, the position of the curve cannot simply discriminate the neutral and non-neutral mutant clones, although a trend of increasing proportions of non-neutral mutations at larger clone sizes is observed in both simulations (figure 6*a*,*b*) and *in vivo* experiments [5]. Neutral mutations hitchhiking on non-neutral clones may grow to large sizes, meaning that analysis restricted to synonymous mutations and mutations in non-expressed genes (Methods), which are not identified as under selection by d*N*/d*S* analysis [5], may not reflect purely neutral dynamics (figure 6*c*). This raises a question of how to meaningfully interpret clone sizes observed in a tissue. This is an important question as there remain a small number of metrics for assessing the neutrality of mutations. While our results have demonstrated the risks of one specific interpretation of the LFIM, they also highlight the dangers of relying on a single measure of neutrality, especially if the underpinning mathematical assumptions are under-explored.

In the specific case of the eyelid data, the original conclusion of non-neutral competition was supported by d*N*/d*S* analysis. While this is a widely used tool, this type of analysis is sensitive to the mutation model used for the neutral hypothesis [26], and detection of positive selection may be unreliable for some types of mutations in some genes. For example, almost all protein-truncating mutations inactivate the protein in which they occur. By contrast, missense mutations in some locations may reduce protein function, while in others, they may generate a constitutively active mutant [30]. In aggregate, these effects may result in a d*N*/d*S* ratio close to 1.

Given the limitations of individual methods to assess neutrality, we speculated that combining discrete approaches may be more informative. To explore this, we directly compared observed clone sizes and the associated d*N*/d*S* ratios of mutations in specific genes. We selected nonsense mutations from a panel of five mutated genes that were identified as under the strongest positive selection in normal oesophagus and which have well-characterized roles in cancer (*TP53*, *NOTCH1*, *NOTCH2*, *NOTCH3* and *FAT1*) (figure 6*d*). For four genes, there is the expected relationship between clone size and selection; that is, mutations in genes under greater selection pressure grow into larger clones. However, *NOTCH2* clones are under selection according to d*N*/d*S* criteria but have a similar size to synonymous clones.

There are multiple possible explanations for this unexpected result for *NOTCH2.* The d*N*/d*S* ratio indicates mutations that promote clonal expansion to a sufficient size to be detected. However, the impact of a mutation on clonal behaviour may alter over time. This may occur if an initial expansion of a mutant clone increases the local cell density. If mutant cell proliferation is sensitive to this change of environment, the rate of clonal expansion may slow. Another potential mechanism is that the mutant clones grow initially due to an advantage over wild-type cells, but are later constrained by the growth of neighbouring clones as the tissue is mutated over time. Both of these behaviours could lead to a high d*N*/d*S* ratio with only a modest increase in clone size, and are similar to observations of a *Trp53* missense mutation in mouse epidermis, where mutant clones have a strong competitive advantage over wild-type cells, but their expansion is constrained [18].

The reverse observation, large clone sizes accompanied by only a modest d*N*/d*S* ratio, may indicate that mutations in a small region or hotspot in the gene can lead to extensive clonal expansion, but mutations in the rest of the gene are under weaker selective pressure. An example from the oesophagus data is *PIK3CA*, which has the largest median clone size of the 14 genes found to be under positive selection in human oesophagus, largely due to the multiple large clones of the hotspot mutation H1047R. This highlights the importance of not just considering the gene in which a mutation occurs, but also the location of the mutation in the structure of the protein. Where large-scale experiments have been performed, more than half of all non-synonymous point mutations in T4 lysozyme fail to substantially effect protein function [31], and mutations in the P53 DNA-binding domain were found to have a ‘broad phenotypic spectrum’ [32]. It follows that using the structure–function relationship to interpret and confirm the mechanism of frequently observed point mutations would support analysis and understanding of such datasets. Other factors such as epistatic interactions with other mutations [33] or age-related changes to the tissue microenvironment [34] could also lead to plastic and context-dependent mutant clone behaviour and a complex relationship between d*N*/d*S* ratios and clone sizes.

## 3. Discussion

We have presented two complementary explanations to resolve the apparent paradox regarding the dynamics of mutant clones in normal human eyelid skin. Both show how non-neutral competition can be consistent with a straight-line LFIM of the inferred mutant clone size distribution—previously claimed to be an indication of neutral competition. Therefore, the mutant clone sizes observed in the normal human eyelid no longer appear to contradict the range of studies that suggest a number of mutations can drive non-neutral expansion of mutant clones in epithelia. We have also shown the benefits of using multiple orthogonal approaches to infer clone behaviour. Finding a consensus can provide a high degree of confidence in the analytical conclusions, and inconsistencies may reveal an issue with one of the methods or help to identify interesting outliers in the data.

We have found that by considering spatial constraints of the tissue, non-neutral simulations can produce a straight-line LFIM, providing a counter example to the proposition that a straight-line LFIM implies neutral competition. We have also shown how the experimental method used to measure the sizes of mutant clones in the eyelid could hide signs of non-neutrality in the clone size distribution. Using isolated single samples which are too small in relation to the clone sizes will lead to underestimation of the size of a significant proportion of clones, as occurred in the eyelid experiment, and could lead to an apparently neutral clone size distribution. However, using over-large samples will reduce the ability to detect smaller clones, which can also lead to a straight-line LFIM because only the largest mutant clones are observed [23] (figure 5*a*). By using a grid of adjacent samples, the larger clones can be more accurately measured without compromising the detection of smaller clones, and can reveal the signs of non-neutrality that would otherwise have been hidden.

We have discussed the effects of sampling in detail. However, there are other potential confounding factors that could appear during DNA sequencing experiments. For example, comparing clone size distributions between genes may be hindered by variations in read coverage and the frequency of sequencing errors across genes, which could lead to different detection limits for small clones in different genes, and therefore different average clone sizes. Furthermore, multiple independent but identical mutations within the same sample would be observed as a single clone (although this is likely to be rare [11]) and some large clones may be caused by somatic mosaicism rather than positive selection [6].

We conclude that mathematical and *in silico* models will be important tools for understanding clonal competition in pre-cancerous tissues. However, difficulties lie in the complex ways in which mutants grow. Specific mutations may increase cell fitness through multiple mechanisms specific to the individual gene function. In Notch1, for example, missense mutations inactivate the protein by disrupting the Notch1–Jagged interface, or through the loss of protein-stabilizing disulfide bonds [5]. Equally, truncating mutations, or the introduction of new splice sites, may reduce the expression levels of functional protein in the cell. Hotspot mutations also demonstrate that the effects of mutations can also vary hugely within the same gene. Mutants may interact in complex ways with other mutants as neighbours or within the same clone and may even depend on the order in which the mutations appear [35,36]. The behaviour of the mutant clone can also change over time, reacting to a changing local environment that may be altered by the mutant itself [18]. Exploring the consequences of adaptive mutant behaviour, while still using models which are simple enough to fit to data and interpret, will be an ongoing challenge in the work ahead.

## 4. Methods

### 4.1. Simulations

The simulations were carried out on a 500 × 500 hexagonal lattice whose edges were wrapped to form a torus. Each cell was assigned a fitness value of 1 at the start of a simulation. Similar to a Moran process [27], one cell was randomly selected at each simulation step to differentiate (was removed from the simulation) and a neighbouring cell was selected to divide to fill the space, with fitter cells having a higher chance of dividing (figure 1*f*). During each division, there was a chance that a mutation would occur in the new cell. If the cell did not mutate during division or if the mutation was neutral, the new cell produced would inherit the fitness of its parent cell. If the mutation was non-neutral, a random value drawn from a normal distribution, *N* ∼ *N* (mean = 0.1, std = 0.1), was added to the fitness of a cell. We show in the electronic supplementary material that our particular choice of distribution does not affect the conclusions of the analysis.

Estimates of cell cycle time in human tissues are hard to verify. However, we did not fit simulations to data, only demonstrated general properties of the models; hence, the exact division rates used do not affect the conclusions of the analysis. For the neutral simulations, we used a division rate of 0.5 per week as estimated from the LFIM of clones in the human eyelid under the assumption of neutral competition [11]. In the non-neutral simulations, the fittest clones can expand much faster than neutral, and therefore, we reduced the division rate to 0.033 per week, so that maximum mutant clone sizes were similar to the neutral simulations. The simulations ran for 3000 weeks (approx. 58 years).

The somatic mutation rate for human tissue has been estimated at approximately 10^−9^ mutations per base pair per cell division [37], although we note that exposure to UV or mutagenic agents (such as stomach acid and alcohol) may substantially alter the mutation rate. With roughly 10^6^ bp included in the targeted sequencing experiment [4], this leads to a mutation rate of 10^−3^ mutations per cell division which we use for the neutral simulations. We use a higher mutation rate of 1.5 × 10^−2^ mutations per cell division for the non-neutral simulations so that the total clone numbers were similar in the neutral and non-neutral simulations.

Clone sizes were defined by the number of cells containing each mutation at the end of the simulation.

### 4.2. Biopsy and sequencing simulations

Biopsies were simulated by taking 25 non-overlapping 70 × 70 cell squares from each grid. Assuming a density of basal cells of 10 000 mm^−2^ [38] and that half of basal cells are progenitors, this would make our biopsies approximately 1 mm^2^, similar in size to those used in the human eyelid [4].

Small clones may only appear in a very small proportion of sequenced DNA reads (if any) and are therefore hard to distinguish from sequencing errors [29], meaning they are not successfully detected as somatic mutations. To replicate this, we assumed a constant 1000× read depth and a requirement of 10 reads as a minimum to observe the mutant. For each mutant, we had a true frequency *f*, the proportion of cells which contained the mutant. We assumed all mutations were heterozygous, so the true VAF was given by 0.5*f*. Each read then had a 0.5*f* chance of containing the mutation, so the total number of mutant reads observed, reads_obs_, was given by a draw from a binomial distribution with *n* = 1000, *p* = 0.5*f*. If reads_obs_ was greater than 10, we recorded the mutant as having a VAF of reads_obs_/1000, otherwise the mutant was unobserved and not included in the results.

### 4.3. First incomplete moment test

We used the first incomplete moment as defined in [11]

$$\mu_{1}(n,t)=\frac{1}{\sum_{m=1}^{\infty }mP_{m}(t)}\sum_{m=n}^{\infty }mP_{m}(t),$$

where $P_{m}(t)$ is the proportion of surviving clones that have *m* cells at time *t*. Normalization using average clone size, $\sum_{m=1}^{\infty }mP_{m}(t)$, means that $\mu_{1}(n,t)=1$ for all values of n smaller or equal to the smallest observed clone size. As in previous studies [11,23], we used *R*^2^, the coefficient of determination, to assess whether the log of the first incomplete moment was a straight line. The line fitting was constrained to pass through the point (*m*, 1), where *m* was the smallest observed clone size.

### 4.4. d*N*/d*S* ratio

Neutral and non-neutral mutations were introduced into the simulations with a known ratio, *a*. d*N*/d*S* was calculated as follows:

$$\frac{dN}{dS}=\frac{N}{aS},$$

where *N* was the number of observed non-neutral clones and *S* was the number of observed neutral clones.

### 4.5. RNA expression

RNA levels for genes in the human oesophagus were obtained from RNA-seq data from the Human Protein Atlas [39] (available from www.proteinatlas.org, accessed 1 October 2018). We used these data to select a set of genes that are not expressed and are therefore highly unlikely to be selected for. It is not clear at which transcripts per million (TPM) value a gene would have sufficient expression to make selection possible. We therefore used a conservative threshold of 0.0 TPM. The genes with 0.0 TPM in the gene panel sequenced in the oesophagus [5] were *ADAM29*, *GRM3*, *KCNH5*, *MUC17*, *PTPRT*, *SCN11A*, *SCN1A* and *SPHKAP*. All genes under positive selection in the human oesophagus mutation data [5] have a non-zero TPM (table 1).

**Table 1.** RNA levels for genes under positive selection in the oesophagus. RNA-seq data from the Human Protein Atlas [39] (Methods) for the genes under positive selection in the human oesophagus [5].

Gene	TPM
NOTCH1	4.1
NOTCH2	22
NOTCH3	45.3
TP53	32.4
CUL3	60.1
FAT1	14.9
ARID1A	19.7
KMT2D	12.6
AJUBA	12
PIK3CA	7.3
ARID2	7
NFE2L2	267.1
TP63	60.9
CCND1	78.9

## Supplementary Material

Supplementary text

## Data accessibility

Source code is accessible via Zenodo at doi:10.5281/zenodo.2839478.
